# Nonvisual Multisensory Impairment of Body Perception in Anorexia Nervosa: A Systematic Review of Neuropsychological Studies

**DOI:** 10.1371/journal.pone.0110087

**Published:** 2014-10-10

**Authors:** Santino Gaudio, Samantha Jane Brooks, Giuseppe Riva

**Affiliations:** 1 Centre for Integrated Research (CIR), Area of Diagnostic Imaging, Università “Campus Bio-Medico di Roma”, Rome, Italy; 2 Department of Psychiatry and Mental Health, Groote Schuur Hospital, University of Cape Town, Anzio Road, Observatory, Cape Town, South Africa; 3 Department of Neuroscience, Uppsala University, BMC, Uppsala, Sweden; 4 Applied Technology for Neuro-Psychology Lab, Istituto Auxologico Italiano, Milan, Italy; 5 Department of Psychology, Università Cattolica del Sacro Cuore, Milan, Italy; University of Udine, Italy

## Abstract

**Background:**

Body image distortion is a central symptom of Anorexia Nervosa (AN). Even if corporeal awareness is multisensory majority of AN studies mainly investigated visual misperception. We systematically reviewed AN studies that have investigated different nonvisual sensory inputs using an integrative multisensory approach to body perception. We also discussed the findings in the light of AN neuroimaging evidence.

**Methods:**

PubMed and PsycINFO were searched until March, 2014. To be included in the review, studies were mainly required to: investigate a sample of patients with current or past AN and a control group and use tasks that directly elicited one or more nonvisual sensory domains.

**Results:**

Thirteen studies were included. They studied a total of 223 people with current or past AN and 273 control subjects. Overall, results show impairment in tactile and proprioceptive domains of body perception in AN patients. Interoception and multisensory integration have been poorly explored directly in AN patients. A limitation of this review is the relatively small amount of literature available.

**Conclusions:**

Our results showed that AN patients had a multisensory impairment of body perception that goes beyond visual misperception and involves tactile and proprioceptive sensory components. Furthermore, impairment of tactile and proprioceptive components may be associated with parietal cortex alterations in AN patients. Interoception and multisensory integration have been weakly explored directly. Further research, using multisensory approaches as well as neuroimaging techniques, is needed to better define the complexity of body image distortion in AN.

**Key Findings:**

The review suggests an altered capacity of AN patients in processing and integration of bodily signals: body parts are experienced as dissociated from their holistic and perceptive dimensions. Specifically, it is likely that not only perception but memory, and in particular sensorimotor/proprioceptive memory, probably shapes bodily experience in patients with AN.

## Introduction

Anorexia Nervosa (AN) is the psychiatric disorder with the highest rate of mortality [1], the onset mainly occurring in adolescent girls and young women. AN is characterised by extremely low body weight and an obsessive fear of gaining weight [2]. Currently, the etiology of AN is not fully understood and there are neither specific nor widely accepted pharmacological or psychological treatment protocols [3]. As confirmed by DSM V [2] criteria AN is characterized by distorted body image with a pathological fear of becoming fat: AN patients perceive their body or body parts as being too fat, even if they are in a severely emaciated state. Several studies have shown that body image distortion (BID) can be considered as a risk factor in the development of AN [4], [5] and its persistence may be among the most important predictors for clinical severity of AN [6].

Behavioural and neuropsychological research considers BID as a multidimensional symptom comprising different dimensions (for e review see [7], [8]), that seem to have specific neural correlates in AN patients [9], [10]. The most widely accepted components remain the perceptive, the affective, and the cognitive [7]. The perceptive component comprises the identification and estimation of one's own body and it refers to the accuracy of the individuals' evaluation of their size, shape, and weight relative to their real proportions [11], [12]. The affective component mainly comprises feelings that individuals develop towards their body's appearance and satisfaction/dissatisfaction of one's own body [13]. Finally, the cognitive component mainly comprises beliefs concerning body shape and appearance and the mental representation of one's own body [13]. Overall, literature on those with AN emphasised that all three dimensions are affected, and that these patients show an overestimation of their body size, a greater body dissatisfaction, and greater self-ideal discrepancies [7].

It is well known that AN patients show an altered interoception, that is, the feeling of the body (e.g. as in hunger, tactile discomfort), which is also involved in body image disturbances [3], and some further studies investigated visuo-proprioceptive integration and tactile impairment of body perception in AN [14]–[16]. However, to date the majority of both behavioural and functional neuroimaging studies, that have investigated body image distortion in AN patients, have used visual tasks and have considered body perception as a unisensory concept linked to the visual domain [7], [8], [10]. Thus, to date the body overestimation and dissatisfaction of AN patients is mainly considered as visual related. However, other senses, as well as the integration of different sensory inputs have been insufficiently considered in the etiology of BID in those with AN.

Nevertheless, it is well known that body perception in general is multisensory and is composed of different inputs: visual, tactile, proprioceptive, and interoceptive [17]. The perception and knowledge of one's own body is due to co-perception and integration of the different sensory information (visual, tactile, proprioceptive and interoceptive) that enables an individual to gain an accurate perception of one's own body [17]. To date, the perception of one's own body, its neural bases, and the integration of different body sensory inputs in healthy subjects is yet to be fully understood [17]–[19]. Furthermore, the terms and the concepts used to refer to one's own body perception or body representation (that is linked/related to the perceptual inputs) are various, and different authors may even use them with contrasting meanings [17], [19]. The two most widely used terms are: “Body schema” and “ body image” [17], [19]. Body schema is a sensorimotor representation of the body that guides actions on the basis of tactile, kinesthetic, visual and labyrinthine inputs. It is elicited by action, regardless of whether the latter is imagined, anticipated and/or executed [19], [20]. The concept of body image is more complex and concerns not only perceptual representations of the body but also emotional and conceptual aspects [19]. However, the debate on how many separate body representations can be identified is open and some authors suggest that “the inconsistence and variability in definition make a good case for giving up the terms body image and body schema completely” [17]. In this review we will not consider the debate on the dichotomy between “body schema” vs “body image”, but rather we will incorporate these domains into our review, and how they relate to BID in those with AN.

Within this framework, the aim of our paper is to systematically review neuropsychological studies that have investigated body perception in AN patients, using the different sensory stimuli, and to classify them on the basis of the different sensory stimuli. In order to do this, we will consider body perception as composed of different perceptual inputs (i.e. visual, tactile, proprioceptive, interocepitve) [17]. The large literature and the presence of several reviews on visual perceptual body-size distortion in AN patients [7], [8] led us to the decision not to collect and review studies that only used visual tasks. Thus, here we conduct a systematic review of neuropsychological studies on AN patients using an integrative nonvisual multisensory approach to body perception. Finally, we will also discuss these findings in the light of AN neuroimaging evidence.

## Method

**Figure 1:**
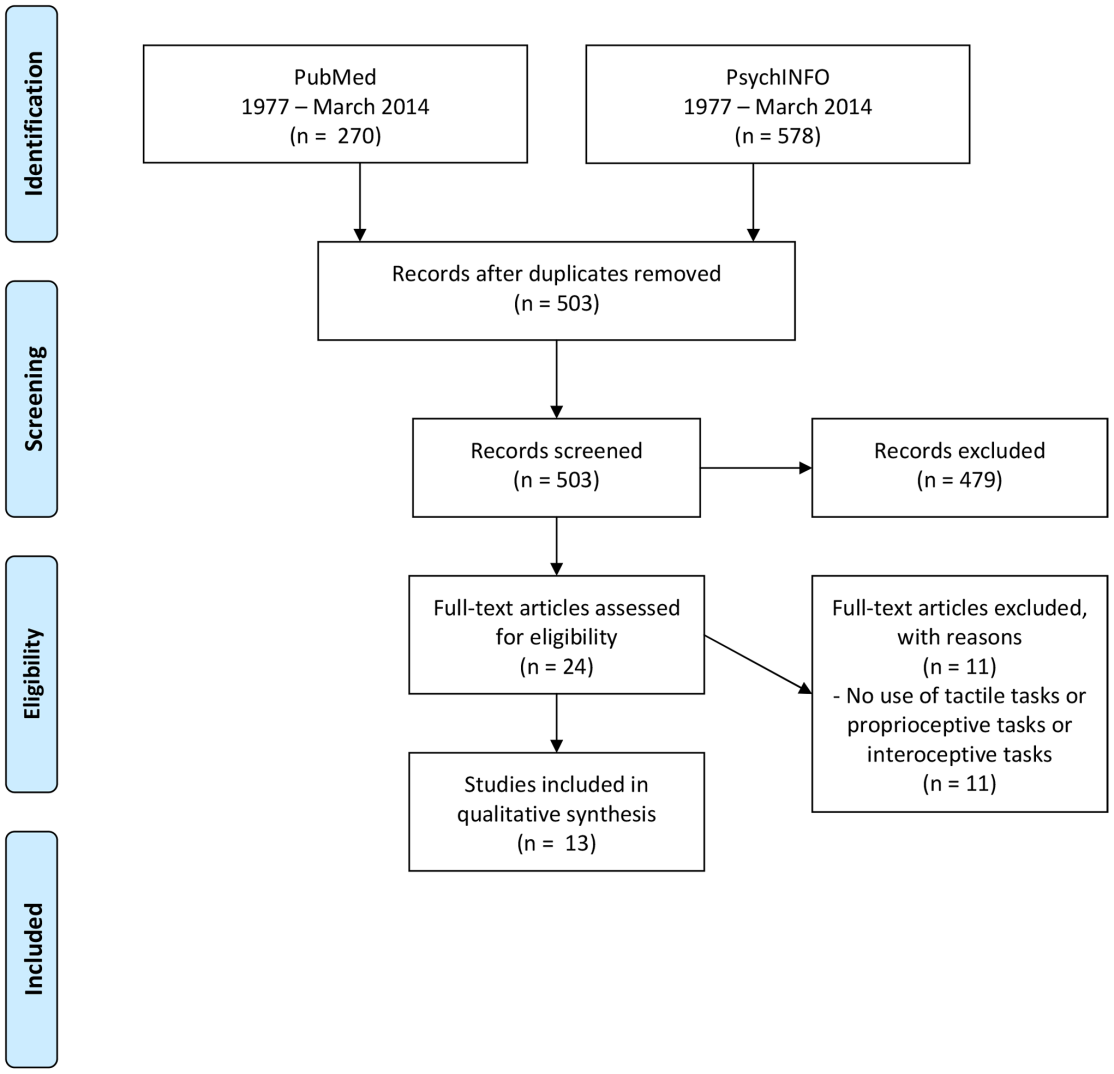
Flow diagram of article selection.

We followed the Preferred Reporting Items for Systematic Reviews and Meta-Analysis (PRISMA) guidelines [21]. The statement consists of a checklist of recommended items to be reported (Table S1) and a four-step flow diagram (Figure 1).

### Search strategy and inclusion criteria

Databases used for the search were: PubMed (1977 to March 2014) and PsycINFO (1977 – to March 2014). We searched using the terms: “anorexia nervosa” or “eating disorders” AND “tactile/touch” or “proprioception/proprioceptive” or “interoception/interoceptive” or “body schema” or “body perception”. The reference lists of examined full-text papers were scrutinised for additional relevant publications. In addition, expert colleagues in the field were contacted for suggestion on further studies not considered in our search.

To be included in the review, studies were required to: a) be written in English, b) investigate a sample of patients with AN or a sample of recovered AN patients in cross-sectional case-control or longitudinal design, c) use tasks directly related to the body of subjects included in the experiment, d) use tasks that directly elicited one or more sensory domains. Studies using tasks that only elicited visual domain or elicited beliefs concerning body shape and appearance (i.e. cognitive component of body image distortion [7]) or used tasks not directly related to the body (e.g. oral object-size evaluation) were excluded. Due to limited number of peer-reviewed studies on body perception in AN, we did not consider confounding factors such as the presence of psychiatric comorbidity, the sample inhomogeneity (e.g. AN subtypes), and treatment history, that may limit some studies included, and the conclusions drawn.

### Classification criteria of neuropsychological studies

We consider body perception as composed of different sensory inputs: proprioceptive, tactile (divided into tactile stimuli and haptic stimuli [see below]), visual, and interoceptive [17]. Accordingly, the classification of studies was based on the different sensory inputs elicited by experimental task designs. We conceptualized the degree of involvement of each sensory input as “primary” (i.e. elicited by the task and predominantly involved), “secondary” (i.e. related but no primary involved: e.g. for the “tactile” sub-component of the tactile stimuli in the presence of a specific haptic task [22] or temperature stimuli [23], [24]) or “absent” (i.e. for visual domain if participants were blindfolded or with eyes closed; for haptic component if participants don't actively explore an object [22]; for tactile component if there were not any tactile stimulus [25]). Tactile input was considered present when the task was based on a tactile stimulus touching the skin [25]. Haptic input was considered as separated from tactile inputs: it was considered present when the task was based on active haptic exploration of objects (e.g. palpate an object) [22]. Proprioceptive input was considered present when the task was based on a sensory judgment about limb and body position [26]. Interoceptive input was considered present when the task tested the sensitivity to visceral activity [27]. Visual input was considered present when the task was based on the viewing of real own body images [28], [29].

### Quality assessment and data abstraction

To reduce a risk of bias, PRISMA recommendations for systematic literature analysis have been strictly followed. Two authors (S.G. and G.R.) independently selected paper abstracts and titles, and analyzed the full papers that met the inclusion criteria, resolving disagreements through consensus. Data extracted from each study were: Degree of involvement of the sensory component of body perception, sample type, study design, sample size, and selected findings.

## Results

Thirteen studies on AN patients were included in the review (Figure 1 and appendix S1). Table 1 reports the sample characteristics of each AN study. Table 2 reports the classification of all studies based on the different sensory stimulus involved in body perception. The degree of involvement of the different sensory stimuli of body perception is estimated depending on the design of the tasks/paradigms used. In subsequent paragraphs the results of included studies for each sensory component are reported.

**Table 1:** Sample characteristics of neuropsychological studies.

Study	Participants	Age (years)		BMI		ED duration (years)	
		Mean	(SD)	Mean	(SD)	Mean	(SD)
*AN subjects cross sectional studies*							
Keizer et al. (2011) [15]	AN: n = 20^a^	22.30	(3.01)	18.54	(2.03)	8.4*	(6.5)
	HC: n = 25	21.32	(2.19)	21.43	(1.77)	-	-
Keizer et al. (2012) [16]	AN: n = 25^b^	24.16	(4.24)	18.96	(2.18)	7.96*	(8.34)
	HC: n = 28	22.54	(2.52)	21.30	(1.76)	-	-
Guardia et al. (2012) [37]	AN: n = 25	24.3	(6.4)	15.14	(1.5)	5.3	(4.8)
	HC: n = 25	23.04	(5.98)	21	(1.99)	-	-
Guardia et al. (2013) [34]	AN: n = 25	22.24	(8.6)	14.89	(1.10)	4.57	(6.52)
	HC: n = 25	22.88	(3.63)	21.65	(1.72)	-	-
Goldzak-kunik et al. (2012) [23]	AN: n = 11	15.8	(0.34)	17.2	(0.50)	-	-
	HC: n = 11	15.0	(0.48)	19.4	(0.58)	-	-
Case et al. (2012) [14]	AN: n = 10	29.1	(11.0)	17.1	(0.9)	36*°	-
	HC: n = 10	25.8	(9.0)	21.7	(1.6)	-	-
Eshkevari et al. (2012) [43]	AN: n = 36	23.0	(18)	16.1	(2.71)	6.0	(11)
	BN: n = 22	22.5	(10)	20.9	(4.28)	7.0	(4)
	EDNOS = 20	27.5	(16)	19.7	(5.54)	11.5	(12)
	HC: n = 61	24.0	(7)	21.5	(2.80)	-	-
Pollatos et al. (2008) [49]	AN: n = 28	21.4	(4.8)	16.6	(1.2)	2.5	(3.2)
	HC: n = 28	22.4	(2.4)	21.4	(4.3)	-	-
*AN subjects longitudinal studies*							
Grunwald et al. (2001a) [22]	AN: n = 10	16.34	(1.30)	15.16	(1.45)	-	-
	HC: n = 10	17.30	(1.25)	22.44	(3.23)	-	-
Grunwald et al. (2001b) [32]	AN: n = 10	15.90	(1.97)	15.24	(1.27)	14.5*	(5.7)
	HC: n = 10	16.14	(0.74)	22.16	(3.01)	-	-
Grunwald et al. (2004) [33]	AN: n = 10	15.90	(1.97)	15.24	(1.27)	14.5*	(5.7)
	HC: n = 10	16.14	(0.74)	22.16	(3.01)	-	-
Epstein et al. (2001) [30]	AN: n = 20	-	-	15.63	(2.43)	-	-
	HC: n = 20	-	-	21.08	(2.29)		
*Recovered AN subjects cross sectional studies*							
Strigo et al. (2013) [24]	REC AN: n = 12	29.7	(6.8)	21.9	(1.65)	-	-
	HC: n = 10	24.8	(6.1)	21.9	(0.73)	-	-

Note: BMI =  Body mass index. n =  number. AN =  anorexia nervosa. HC =  healthy control. BN =  bulimia nervosa. EDNOS  =  eating disorder not otherwise specified. REC-AN  =  recovered from AN.

* =  months.

° =  median.

a The sample was composed by: AN: n = 15; AN subtype EDNOS: n = 5.

b The sample was composed by: AN: n = 11; EDNOS: n = 14.

**Table 2:** Classification of neuropsychological studies based on the multisensory model of body perception in AN.

Study	Index condition	Degree of involvement of the sensory component of body perception				
		Tactile		Proprioceptive	Interoceptive	Visual
		Tactile	Haptic			
Grunwald et al. (2001a) [22]	Haptic exploration of “sunken reliefs” (blindfolded) and to reproduce the structure of the stimuli	*	**	*	*	-
Grunwald et al. (2001b) [32]	Haptic exploration of “sunken reliefs” (blindfolded) and to reproduce the structure of the stimuli	*	**	*	*	-
Grunwald et al. (2004) [33]	Haptic exploration of “sunken reliefs” (blindfolded) and to reproduce the structure of the stimuli	*	**	*	*	-
Keizer et al. (2011) [15]	Tactile estimation task	**	-	*	*	-
	Distance comparison task	-	-	-	-	**
Keizer et al. (2012) [16]	Tactile estimation task	**	-	*	*	-
	pressure detection task and two point discrimination	**	-	*	*	-
Guardia et al. (2012) [37]	manually set a rod to investigate the effect of passive lateral body inclination (i.e. three postural conditions) on the subjective vertical	*	_	**	*	-
Guardia et al. (2013) [34]	Haptic exploration of 10 cubes (blindfolded) and to judge whether the variable stimulus was smaller than the standard.	*	**	*	*	-
	Visual exploration of 10 different square and to judge whether each variable stimulus was smaller than the standard stimulus.	-	-	*	*	**
	manually set a rod (under tactile and visual conditions) to investigate the effect of passive lateral body inclination (i.e. three postural conditions) on the subjective vertical.	*	_	**	*	*
Goldzak-kunik et al. (2012) [23]	View 7 different schematic body-shape silhouettes of adolescent girls	-	-	*	*	**
	order by size (blindfolded) 10 beads that were individually handed to them by feeling them with their hands.	*	**	*	*	-
	Identify (blindfolded) 8 shapes (e.g. egg, disk, heart, star, etc) that were individually handed to them by feeling them with their hands.	*	**	*	*	-
	“Participants were blindfolded and asked to estimate the relative height of each hand holding a vertical handle placed in the sloping rails of an apparatus at approximately chest height.”	*	*	**	*	-
	Ice cube in a plastic bag was placed in the center of the back of the right hand. Patients could remove the ice if they felt uncomfortable	*	_	*	**	-
Case et al. (2012) [14]	a size–weight illusion battery	*	**	**	*	**
Eshkevari et al. (2012) [43]	rubber hand illusion paradigm	**	-	**	*	**
Pollatos et al. (2008) [49]	heartbeat perception task	-	-	*	**	-
Epstein et al. (2001) [30]	Proprioception test	*	-	**	*	-
	Finger localization test	**	-	*	*	*/-
	Right-left orientation test	*	-	**	*	*/-
Strigo et al. (2013) [24]	Pain temperature stimuli	*	-	*	**	*

Note: Degree of involvement of the sensory component of body perception: ** =  present; * =  related but no primary involved; - =  absent. Brief description of sensory components of body perception: Tactile  =  the tasks was based on tactile stimulus touching the skin; Haptic: the tasks was based on active haptic exploration of objects (e.g. palpate an object); Proprioceptive  =  the task was based on a sensory judgment about limb and body position; Interoceptive  =  the task tested the sensitivity to visceral activity;

Visual: the task was based on the viewing of real own body images.

### Tactile perception in AN

Tactile perception is related to the processing of a stimulus touching one's own body and is processed in several modalities [25]. In particular, the size and shape of an elementary tactile stimulus touching the skin involves size, shape, configuration, and posture of the body. In our review we consider tactile perception as the perception of an external stimulus touching the skin [25] and we only discuss studies that used tactile stimuli on AN patients.

Three studies have investigated the alteration of tactile perception in AN patients using different paradigms [15], [16], [30].

Epstein et al. [30] longitudinally studied a sample of AN inpatients compared to a control group using three different tests: proprioception test, finger identification test and right and left orientation test. Particularly, the finger identification test was mainly composed of tactile inputs and it comprised three parts: patients had to recognize (1) which finger was being touched while their eyes were open, (2) while their eyes were closed, and (3) which pairs of fingers were being touched while their eyes were closed. These authors found that AN patients in pre-treatment evaluation showed significantly lower scores in part 3 of the test compared to controls. No differences were found in post-treatment evaluation between AN patients and controls.

Keizer et al. [15] used two different tasks in a sample of AN patients and in a control group in order to investigate tactile perception (the “tactile estimation task”) and “visual body representation” respectively. In the first task, participants were blindfolded and the researcher pressed two pointers of a caliper simultaneously and lightly on the skin (the distance between the two pointers was set at three different distances). The authors tested two body parts and distinguished them between sensitive (i.e. the abdomen) and insensitive (i.e. the arm) body areas. During the task, participants were asked to indicate the distance between two tactile stimuli (the distance was estimated by varying the separation between their right thumb and index finger). The second task was the “Distance Comparison Task”: 28 word-pair (each word-pair consisted of two identical body parts, representing left and right side of the body), distinguished between sensitive (es. hips) and insensitive (es. ears) body parts, were presented to the participants; subsequently, participants were asked to estimate whether the last presented word-pair reflected a smaller or larger distance on their own body than the first presented word-pair. Here we will mainly consider the first tactile task. The tactile estimation task showed that AN patients overestimated the size of tactile distances, with no difference between sensitive and insensitive body parts. They also found that a high score of body dissatisfaction was related to more severe disturbance in tactile distance estimation. At the same time, the study confirmed that AN patients showed an inappropriate visual mental image of their body.

Keizer et al. [16] investigated tactile perception in AN patients in a subsequent paper. In this work, the authors used the previous reported “tactile estimation task” and two measures of elementary tactile perception in a sensitive (i.e. abdomen) and an insensitive (i.e. arm) body part. The first elementary tactile task was focused on detection of pressure provided by a single stimulus applied to the skin, using the Von Frey task [31]. Participants were blindfolded and they were asked to indicate whether or not they perceived a stimulus. The second task was the “two point discrimination” task that assessed tactile acuity: the evaluation of the minimum distance that was needed between two tactile stimuli for a participant to report feeling pressure from two distinct stimuli. The results of the “tactile estimation task” confirmed the results of their previous study, showing that AN patients made larger distance estimations than controls on both arm and abdomen. The results of the pressure detection task revealed that AN patients showed a lower pressure detection threshold on their abdomen compared to controls. At the same time, AN patients and controls showed similar tactile detection thresholds for their arm. The results of the two point discrimination task showed that AN patients had a higher discrimination threshold compared to controls, with no differences between the arm and the abdomen.

In summary, the studies that investigated tactile component of body perception in AN found that AN patients showed alterations in tactile perception from basic to complex tactile tasks [15], [16], [30]. Thus it may be assumed that the tactile domain can have a role in body image distortion in AN.

### Haptic perception in AN

Haptic tasks are different from tactile stimuli in that they required an active explorative movement of the exploring limb [22]. The resulting changes in receptors of the skin, muscles, tendons, and joints lead to successive information about the explored object [22]. Five studies have explored haptic perception in AN patients [22], [23], [32], [33], [34]. The first three studies of haptic perception of AN patients were made by Grunwald and colleagues [22], [32], [33] using a longitudinal design (before and after weight gain). AN and control subjects were asked to keep their eyes closed and to palpate 12 or 6 individual “sunken reliefs” (presented to participants in random order). Following the haptic task, subjects were asked to reproduce the structure of the stimuli. The first study [22] showed that AN subjects have greater difficulty with complex haptic information than controls. For example, AN subjects showed a longer exploration time for haptic stimuli and a lesser quality of reproductions of complex haptic stimuli. Interestingly, AN subjects also showed a poorer quality of reproductions of haptic stimuli after weight gain. With regard to this, in their second study Grunwald et al. [32] found similar results compared to their previous study [22]. AN patients showed a significantly poorer quality of reproduction of haptic stimuli compared to controls (with no differences before and after weight gain), but no differences were found in exploration time between the two groups. Furthermore, the authors also recorded a digital electroencephalogram during the haptic task and found theta power differences between AN patients (before and after weight gain) and controls in the right hemisphere and right parietal regions. Grunwald et al. [32], [33] also found a theta asymmetry over central regions during the haptic task in AN patients (both in acute stage of starvation and after weight gain), suggesting an over-arousal of the right hemisphere in AN patients while performing haptic exploration tasks.

A haptic test was used by Goldaz-Kunik and colleagues [23]. In the study, participants were blindfolded and asked to identify 8 simple shapes (e.g. egg, disk, heart, and star). AN patients compared to the control group showed no differences of object sizes and shapes evaluation by manipulation. Finally, a haptic task was also used by Guardia et al. [34] as a preliminary test in order to investigate spatial cognition in AN. In this study, haptic stimuli consisted of 10 cubes (with a side length ranging from 1 cm to 10 cm). Each trial consisted of the presentation of two stimuli and participants manipulated consecutively a standard stimulus (the 5 cm cube) and a variable stimulus. Participants were blindfolded and they had to evaluate whether the variable stimulus was smaller/equal/greater than standard stimulus. The authors did not find any significant difference in haptic discrimination between the AN patients and control group. They suggested that haptic perception is not impaired in the discrimination of simple shapes in AN.

To sum up, haptic reproduction abilities were poorer in AN patients compared to controls both before and after weight gain [22], [32], [33]. Furthermore, these difficulties seems to be related to functional alterations of the right parietal lobe [32], [33]. On the other hand, no significant differences were found between AN patients and controls in the discrimination of simple haptic stimuli [23], [34].

### Proprioceptive perception in AN

Proprioception is the sense of knowing where limb and body position is in space [26]. Control and perception of body orientation is allowed by multiple sensory and motor mechanisms ranging from simple peripheral mechanisms to complex ones involving the highest levels of cognitive function and sensory-motor integration [35]. Between them, an important role is played by spatial orientation constancy, defined as ability to preserve the sense of gravitational and vertical orientation despite inclination of the body and/or visual context [36].

Proprioceptive perception has been investigated in AN patients by four studies [23], [30], [34], [37].

Epstein et al. [30], as reported in the tactile paragraph, longitudinally studied (pre- and post-treatment) a sample of AN inpatients compared to a control group using three different tests. As regards the proprioceptive component of body perception, they used the “proprioception test” and the “right-left orientation test”, that assessed the capacity to locate one's body parts in space and three features of right-left orientation (i.e. “orientation toward subject's own body”, “orientation toward a confronting person”, and “combined orientation toward subject's own body and a confronting person”) respectively. They found that AN patients showed significantly lower scores in the “right-left orientation test” at pre-treatment assessment as compared to controls, while they found no significant differences between the two groups in such test at post-treatment assessment and in the “proprioception test” at both assessments. Goldaz-Kunik et al. [23] found no differences between AN patients and controls using a “kinaesthesia” task to examine sensory dimension relevant to spatial and motion aspects of body-size perception (in particular, blindfolded participants estimated difference in eight of each hand holding a vertical handle placed in the sloping rails of an apparatus).

In a first study, Guardia et al. [37] investigated spatial orientation constancy in AN with an experimental task used in hemineglect patients with right parietal lesions [38]. In the study, participants were asked to manually set a rod, with no visual feedback, into vertical position when the body was upright and when it was tilted. The classic experimental task, which includes both visual and tactile feedback, produces two effects: The A-effect is characterized by deviations of the subjective vertical (i.e. the sum of various weighted vectors representing visual, gravitational and body cues [39], [40]) towards the axis of the head and usually found with vision and large tilts; the E-effect is characterized by deviations of the subjective vertical away from the axis of the head and usually found with tactile adjustment. The authors found that there was no difference between AN patients and control group in an upright condition, while AN patients showed a higher A-effect compared to controls when the body was roll-tilted to the right and left.

In a subsequent paper, Guardia et al., [34] used the same experimental task, but using both visual and tactile modalities. In this paper, the authors found similar results compared to the previous work (i.e. the A-effect yielded by a body tilt was higher in AN patients compared to the control group, whereas no difference between the two groups was observed when participants were in an upright position). Interestingly, the authors also found that altered spatial orientation was observed in both tactile and visual tasks. Under the body Z-axis task, the authors revealed that tactile and visual body Z-axis judgments in the upright position showed no differences between AN patients and the control group. While, tilting led to significant deviations in tactile and visual body Z-axis, with participants judging the body as being more tilted than it really was. Furthermore, the AN group showed a more marked bias towards the tilt. At the same time, the authors found a negative correlation between the A-effect (deviations of the subjective vertical towards the axis of the head) and interoceptive awareness: “the greater the A-effect, the lower the interoceptive awareness”.

In summary, an impaired spatial orientation constancy was found in AN patients compared to controls [34], [37]. Furthermore, within a longitudinal analysis AN patients in the acute state of the disease showed that some proprioceptive abilities resulted poorer compared to controls (i.e. in the “right-left orientation test”) [30]. Yet AN patients at post-treatment evaluation showed an improvement of such proprioceptive abilities [30]. These results suggest an impairment of the proprioceptive component of body perception that may contribute to body image distortion in AN.

### Haptic-visual-proprioception integration in AN: the size-weight illusion

Case et al. [14] used a size–weight illusion battery to evaluate visuo-haptic integration in 10 females with AN and 10 healthy females. Size-weight illusion is a powerful demonstration of the predictive power of visual perception [14]. It consists of the subject experiencing the size–weight illusion when he/she underestimates the weight of a larger object (e.g. a disk) when compared to a smaller object of identical shape and weight. Different studies suggest that a size–weight illusion is the result of some perceptual rescaling based on prior haptic perception, vision, and visual expectation [41], [42]. The authors found that the ability to discriminate weight did not differ between AN patients and control subjects. At the same time, AN patients showed a reduced size–weight illusion compared to controls. In particular, the differences in size–weight illusion between AN patients and control subjects were larger when disks were closer in weight. Furthermore, patients showed a reduced “reverse” size–weight illusion compared to control subjects.

To sum up, this study showed that although the ability of AN patients to discriminate weight was unaltered, they had a significantly reduced SWI compared to controls. These results suggest that AN patients may have a decreased integration of visual and proprioceptive information that can affect body perception.

### Visual-tactile-proprioceptive integration in AN: The rubber hand illusion

Eshkevari and colleagues [43] used the rubber hand illusion (RHI) paradigm [44] to investigate bodily self experiences in ED Patients (i.e. AN, BN and EDNOS) and recovered ED patients. The RHI paradigm is considered a three-way interaction between touch, vision, and proprioceptive perception of body position in space [45], [46] and it is widely used to investigate embodiment in healthy subjects (e.g. Longo et al. 2009 [47]). Eshkevari and colleagues [43] performed the RHI paradigm based on the original version of Botvinick & Cohen [44], using the two classic outcome measures: proprioceptive drift and embodiment score. In particular, to obtain proprioceptive drift participants were asked to indicate where they felt the tip of their left index finger was located prior to and following each visuotactile stimulation (i.e. two visuotactile induction conditions were performed: asynchronous and synchronous). Bias in these proprioceptive judgements towards the fake hand due to visuotactile stimulation is taken as a measure of the visual dominance of the fake hand over proprioception of one's own hand. On the other hand, the score of experience of embodiment over the rubber hand is obtained by the self-report questionnaire that provides a subjective measure of the illusion. Eshkevari and colleagues [43] found that AN patients showed a significantly greater proprioceptive drift as compared to controls. In particular, AN patients showed significantly greater proprioceptive drift in the synchronous vs the asynchronous condition. At the same time, AN patients reported experiencing embodiment significantly more strongly than the control group. In particular, AN patients showed significantly greater embodiment scores in the synchronous vs the asynchronous condition. Similar results were found in the whole eating disorder group examined by the authors. In addition, both proprioceptive drift and embodiment score correlated significantly with ED psychopathology variables (e.g. drive for thinness, body dissatisfaction) in the whole eating disorder group examined.

In summary, this study suggest that AN patients experienced both greater embodiment and greater proprioceptive drift than controls. The greater RHI showed by AN patients compared to controls suggests an alteration in visuo-tactile-proprioceptive integration of body perception in AN.

### Interoceptive perception in AN

Interoception refers to the processing of visceral sensations and internal bodily responses, which are often concomitant with emotional responses [27]. Interoception includes a range of sensations (e.g. heartbeat, perception of temperature, intestinal tension, hunger, pain, anxiety) [48]. It is widely accepted that AN patients show an impairment of interoception and self-awareness [48]. Nevertheless, few studies have directly investigated sensitivity to visceral activity or internal bodily responses [23], [24], [49].

Pollatos et al. [49] studied a sample of AN patients and a control group using a heartbeat perception task: participants had to count their own heartbeats and they were not permitted to take their pulse with medical equipment. These authors found that AN patients showed a poorer heartbeat perception compared to controls. They suggest that heartbeat detection reflects a general sensitivity for visceral processes and that AN patients have a reduced aptitude to accurately recognize bodily signals. Goldzac-Kunic and colleagues [23] also investigated cold pain in AN patients compared to a control group, using an ice cube in a plastic bag as a pain temperature stimulus. They did not show differences between the two groups in cold pain responses; rather, they revealed a trend for greater sensitivity to cold temperature in AN patients. Strigo et al. [24] studied a sample of recovered AN patients and a control group using functional magnetic resonance and an interoceptive task. It was composed of painful heat stimuli preceded by different colour signals that indicated the strength of upcoming heat stimuli. These authors found no differences between the two groups in anticipatory anxiety and heat pain scores. At the same time, they found that AN patients showed greater activation within dorsolateral prefrontal cortex and decreased activation within posterior insula during painful stimulation, and greater activation within right anterior insula, dorsolateral prefrontal cortex and cingulate during pain anticipation as compared to controls.

To sum up according to one study AN patients showed no differences in perception of painful temperature stimuli compared to controls subjects [23]. On the other hand, alterations in neural response to painful temperature stimuli were found in recovered AN patients compared to controls (without differences in temperature perception between the two groups) [24]. Furthermore, a lower capacity to assess interoceptive stimuli (i.e. heartbeat perception) was found in AN patients compared to controls [49]. These results suggest that the interoceptive component of body perception may be affected in AN and can contribute to body image distortion.

## Discussion

This paper is, to our knowledge, the first to systematically review studies that have investigated body perception in AN patients assessing nonvisual sensory stimuli (i.e., tactile, proprioceptive, interoceptive, and visual when not the only elicited sensation). Body Image Distortion (BID) is one of the core symptoms of anorexia nervosa (AN) [2]. It is considered a multidimensional construct that is composed of three main components (i.e. perceptive, affective, and cognitive) and is characterized by body overestimation, body dissatisfaction and greater self-ideal discrepancies [7]. Even if body perception is multisensory [17], the majority of both behavioural and functional neuroimaging studies have mainly focused on body misperception and dissatisfaction of AN patients as visual related unisensory concept [7], [8], [10]. Here we propose a simple nonvisual multisensory model of the impairment of body perception in AN as an integrative view of visual misperception of the body in AN patients.

Overall, we found that 13 studies have investigated nonvisual multi-modal sensory alteration of body perception in AN. AN patients showed alterations in tactile [15], [16], [30], propriocetive [23], [30], [34], [37], and interoceptive [23], [24], [49] components of body perception, as well as in haptic-visual-proprioception integration [14] and in visuo-tactile-proprioceptive integration [43]. Haptic perceptive alterations were only found in AN patients in specific complex tasks [22], [32], [33]. The main evidence of our review is that nonvisual domains of body perception remain under investigated in AN. At the same time the results, though preliminary, consistently show an impairment in each sensory domain of body perception in AN patients.

Regarding tactile perception, the different studies have used both basic (i.e. finger identification test, Von Frey task, and two point discrimination task) [16], [30] and complex tactile tasks (i.e. tactile estimation task) [15], [16]. Abnormalities on tactile perception were found in AN patients in all different levels tested. Regarding complex tasks, AN patients showed an overestimation of the size of tactile distances in the tactile estimation task compared to controls [15], [16]. These authors, having also found an altered visual mental image of the body in AN patients and correlations between their body dissatisfaction levels and tactile and visual body perception, suggested that tactile disturbances in AN patients result from top-down influences of body dissatisfaction on mental body representations involved in tactile size estimation [15]. In this view, lower scores of the most complex task of the finger identification test were found in AN patients prior to treatment but not in post-treatment evaluation compared to controls. Such findings also suggest that lower tactile capacity may be a state-related deficit in executive function rather than a simple impairment of tactile body perception [30]. On the other hand, a bottom-up explanation for tactile disturbances cannot be ruled out. In fact, AN patients showed the same overestimation of the size of tactile distances on both sensitive (i.e. the abdomen) and insensitive (i.e. the arm) body parts compared to controls [15]. From this alternative perspective, taking into account that pressure detection and discrimination between two pressure points on the skin are elementary somatosensory information that can not involve the “mental body representation” [50], lower pressure detection threshold and higher discrimination threshold found in AN patients compared to controls suggests alterations on a basic level of tactile perception in AN patients [16].

It has been suggested that localising a touch on the body surface is a two-stage process [25]: the first consists of a localization of stimulus within a somatotopic map as a purely somatosensory process; the second consists of a localisation onto a corresponding bodily location as a somatoperceptual process, referred to a representation of the body. Interestingly, each stage of the process can determine tactile localisation errors as shown by lesional and transcranial magnetic stimulation studies (for e review see Longo et al. [25]). Accordingly, it can be suggested that both top-down and bottom-up information may play a role in the altered perception and interpretation of the body tactile domain of AN patients.

To date, no studies have investigated tactile perception in AN patients using neuroimaging techniques. Interestingly, Akatsuka et al. [51] examined neural correlates of two similar tactile tasks used in reported AN studies (i.e. two point discrimination task and pressure detection task) in healthy subjects (women and men). They found that the two point discrimination task was related with the activation of the inferior parietal lobe, suggesting that this area plays an important role in such task. Inferior parietal lobe alterations were found in AN patients in both structural and functional studies [52], [53], [54]. In particular, inferior parietal lobe alterations seem to be related to the perceptive component of BID in AN [10]. Although the degree of interaction between tactile and visual inputs in body perception remains somewhat unclear [25], these results lead us to hypothesize that tactile impairment of AN patients could be related to parietal lobe alterations.

Overall, although investigation of the tactile component of body perception in AN patients is still in its early stages, existing results showed abnormalities on tactile perception in both basic and complex tactile tasks and suggest that the tactile domain can contribute to body perception disturbances in AN.

The tactile component also includes haptic perception that is, based on active haptic exploration of objects [22]. AN patients showed no impairment in the identification of simple shapes [23], [34], while they showed greater difficulty with complex haptic information compared to controls both before and after weight gain [22], [32], [33]. The last two studies also revealed that AN patients had electroencephalographic alterations during haptic tasks in parietal areas. These authors suggested that haptic alterations, that are linked to an altered capacity in processing perceptions and somatosensory integrations in AN patients, are related to functional alterations of the right parietal lobe [32], [33]. Given the limited number of studies on haptic perception in AN, new neuropsychological studies with multisensory paradigm, involving the haptic domain, are needed to better define haptic impairment of AN patients and clarify the possible role of such somatosensory ability in BID.

Regarding proprioceptive perception, the studies that explored such component of body perception used specific tasks such as right-left orientation test [30] and investigated spatial orientation constancy using a task previously used on hemineglect patients [34], [37]. These studies found an impairment in the right-left orientation [30] and altered spatial orientation [34], [37] in AN patients compared to controls. Guardia et al. [37] suggested that AN patients showed impaired spatial cognition and proposed that reduced perception of spatial orientation may be related to poor awareness of interoceptive inputs. Interestingly, AN patients showed alterations in posterior parietal areas (e.g. Mohr et al. [9]) that are also related to egocentric spatial reference frame directly involved in spatial cognition [55]. In this view, suggested low capacity of AN patients to integrate ego- and allo-centric spatial frame of references related to posterior parietal areas alterations [56], [57], [58] may explain impaired proprioceptive processing in AN.

Interestingly, the results of AN studies that investigated proprioceptive abilities have some similarities to disorders of visuo–spatial orientation found in neglect patients [38], [59], [60], [61]. Kerkoff [59] found that neglect patients with predominantly parietal lesions showed visual- and tactile–spatial orientation deficits in axes other than the horizontal, suggesting a critical role of the right parietal cortex for perception of visual axis-orientation of vertical, horizontal and oblique orientations. In a more recent study Funk and colleagues [38] assessed spatial orientation in neglect patients, patients with left- or right-sided brain damage without neglect and healthy controls. Their data showed a systematic deviation of the subjective vertical (an increased A-effect) in neglect patients, which can be explained by a stronger attraction of subjective vertical by the idiotropic vector, due to impaired processing of gravitational information. In a later study exploring the same issue, Funk and colleagues [60] found a modulation of subjective visual vertical as a function of frame tilt. This result suggested an increased influence of contextual cues on subjective vertical in neglect patients as a consequence of impaired processing of gravitational information. Utz and colleagues [61] explored the multimodality of these impairments and demonstrated multimodal and multispatial deficits in the judgment of verticality in patients with visuospatial neglect. Moreover, they explained this deficit with an altered representation of verticality caused by lesions of brain areas related to multisensory integration and space representation in the right temporo-parietal cortex.

Overall, proprioceptive alterations found in AN patients suggest an impairment of such component of body perception that can contribute to body image distortion in AN and could be mainly related to parietal alterations found in AN patients.

Regarding multisensory integration, one study investigated visuo-tactile-proprioceptive integration, using the rubber hand illusion (RHI) paradigm [43], and one study investigated haptic-visual-proprioception integration, using size-weight illusion [14]. Eshkevari and colleagues [43] showed that AN patients experienced the RHI more strongly than controls. In particular, both the cognitive measure (i.e. embodiment score) and the perceptual measure (i.e. proprioceptive drift) of RHI were significantly greater in AN patients compared to control subjects. These results suggest that AN patients have increased sensitivity to the visual component of body perception [43]. Interestingly, a subsequent study of the above mentioned group of research examined a sample of patients recovered by mixed eating disorder (20 AN recovered patients were assessed) using the RHI paradigm [62]. In this second study, these authors found that recovered eating disorder patients showed a significantly greater embodiment score compared to controls (i.e. same sample as in Eshkevari and colleagues 2012 [43]). Furthermore, no significant differences in embodiment score were found between recovered eating disorder patients and acute eating disorder group (i.e. same whole eating disorder sample as in Eshkevari and colleagues 2012 [43]). At the same time, regarding proprioceptive drift, the recovered eating disorder patients were not significantly different from both acute eating disorder group and control group [62]. Taking into account that the hand is a body part considered as insensitive in weight and shape evaluations [63], these results suggest that AN patients have a heightened sensitivity to visual information that may be related to an altered multisensory integration or to an impaired somatosensory information processing of the body [43].

The study that explored size–weight illusion in AN patients suggests they have a cross-modal sensory integration deficit with a reduced reliance on visual input in judgments of weight [14]. Specifically AN patients showed a greater reliance on sensorimotor/proprioceptive memory compared to a reduced reliance on visual input in judgments of weight [14]. Apparently, if visual input may influence force predictions during the initial interaction with objects, after some interaction immediate sensorimotor/proprioceptive memory comes to guide such predictions, independent of perceptual cues [41].

Interestingly, reduced size–weight illusion experienced by AN patients has some similarities to impaired size–weight illusion showed by a patient with a large left temporal parietal lesion [64]. This patient also evidenced independent processing of perceptual and sensorimotor predictions in size–weight illusion and lack of an effective sensory integration. This study also suggested that the temporo-parietal cortex may be responsible for such activity, predicting relevant object characteristics both for perceptual judgments and sensory motor processing.

Overall, although multisensory integration of body perception in AN patients has been less investigated, these findings suggest an impaired integration of different sensory signals that may sustain altered multisensory experiences of the body in AN.

Studies that directly investigated interoception in AN patients are few and used heartbeat detection task [49] and temperature stimuli [23], [24]. Overall, although these studies did not show significant differences in the evaluation of temperature stimuli between AN patients and controls [23], they showed an impairment of interoception (i.e. poorer heartbeat detection) in AN patients [49] and an altered neural response to painful heat stimuli in recovered AN patients [24] as compared to controls. Interestingly, altered activation of the insula during the painful heat stimuli seems to confirm the role of the insula in altered detection of the “inner state” of the body in AN patients [48]. At the same time, such results are consistent with the evidence that the insula is involved in the affective component of visual body image distortion in AN [10].

These preliminary data on interoceptive impairment of AN patients suggest that the capacity to accurately recognize bodily signals in both AN and recovered AN patients should be further explored to clarify its role in internal body experiences and body image distortion.

The results of this review seems to enhance our understanding of BID in AN, suggesting a multisensory impairment of body perception that is broader than common visual misperception of AN patients. Moreover, our findings seems to suggest that AN is characterized as a perturbation of the experience of the body at its most primary level. Globally, the different perceptual alterations suggest an altered capacity of AN patients to process and integrate bodily perceptions: body parts are experienced as dissociated from their perceptive dimensions. Specifically, it is likely not only perception but memory, and in particular sensorimotor/proprioceptive memory, probably shapes bodily experience in patients with AN. In other words, AN patients disintegrate physical from subjective dimensions of bodily experience [65]. However, as yet the question remains as to the mechanism or reason for this disintegration.

### Limitations

When considering the findings of our review, it must be borne in mind that we did not consider confounding factors such as the presence of psychiatric comorbidity, sample inhomogeneity (e.g. AN subtypes), and treatment history that may limit our interpretations. Also, as mentioned at the beginning, there is still much debate concerning definitions and sub-definitions of BID. Nevertheless, our review attempts to provide a comprehensive overview and discussion of current neuropsychological and brain imaging findings concerning deficits in body perception, in people with AN. Another limitation of the review is the relatively small amount of literature available in this area. However, all the reported studies have control groups and there is more than one study for each non-visual component of BID: seven of the studies included samples of at least 20 or 25 patients and a matched control group, four studies included samples of 10 patients with a matched control group, and one study included 11 patients with a matched control group; the final study included 12 AN recovered individuals and a matched control group.

### Conclusions and future directions

This systematic review proposed a nonvisual multisensory approach to body perception in AN. Although our review highlighted that nonvisual domains of body perception remain under investigated in AN, the results of our paper, even if preliminary, showed that AN patients had a multisensory impairment of body perception that involve tactile and proprioceptive sensory components, in addition to the well studied visual misperception. Furthermore, impairment of the tactile and proprioceptive components may be mainly associated with alterations of the parietal cortex in AN patients. The interoceptive component and multisensory integration have been poorly explored directly in AN patients.

To sum up, this review leads us to suggest that BID in AN is a pervasive and multisensory disorder that goes beyond visual domain and involves the global experience of the body.

It is beyond the scope of this review to draw conclusions regarding the processes responsible for BID in AN patients. Nevertheless, current results imply the need of novel and focused therapeutic strategies that will include BID within treatment targets, together with cognitions and eating behaviour.

It remains critical to define the complexity of BID in AN. Further research is needed to try to disentangle the role of each sensory component and to understand altered interaction among different sensory inputs in BID in AN, particularly regarding the interoceptive component. This research should use multisensory approaches of body perception in AN patients using specific paradigms and tasks, as well as neuroimaging techniques to explore neural correlates.

## Supporting Information

Table S1 **PRISMA checklist.**
(DOC)

Appendix S1 **Full search data.**
(DOC)
