# Supplementary material for: Nonvisual Multisensory Impairment of Body Perception in Anorexia Nervosa: A Systematic Review of Neuropsychological Studies
Source: PLoS One. 2014 Oct 10;9(10):e110087. doi: 10.1371/journal.pone.0110087 (PMC4193894; doi:10.1371/journal.pone.0110087)
Supplement: Appendix S1 — Full search data. (DOC) [file pone.0110087.s002.doc]

Appendix S1. Full search data

| PubMed |  |
| --- | --- |
| “anorexia nervosa” AND “tactile“  “anorexia nervosa” AND “touch”  “anorexia nervosa” AND “proprioception”  “anorexia nervosa” AND “proprioceptive”  “anorexia nervosa” AND “interoception”  “anorexia nervosa” AND “interoceptive”  “anorexia nervosa” AND “body schema”  “anorexia nervosa” AND “body perception” | 8  15  3  3  7  51  9  16 |
| “eating disorders” AND “tactile”  “eating disorders” AND “touch”  “eating disorders” AND “proprioception”  “eating disorders” AND “proprioceptive”  “eating disorders” AND “interoception”  “eating disorders” AND “interoceptive”  “eating disorders” AND “body schema”  “eating disorders” AND “body perception” | 4  19  5  3  3  94  5  25 |

| PsycINFO |  |
| --- | --- |
| “anorexia nervosa” AND “tactile“  “anorexia nervosa” AND “touch”  “anorexia nervosa” AND “proprioception”  “anorexia nervosa” AND “proprioceptive”  “anorexia nervosa” AND “interoception”  “anorexia nervosa” AND “interoceptive”  “anorexia nervosa” AND “body schema”  “anorexia nervosa” AND “body perception” | 11  30  4  3  7  87  8  35 |
| “eating disorders” AND “tactile”  “eating disorders” AND “touch”  “eating disorders” AND “proprioception”  “eating disorders” AND “proprioceptive”  “eating disorders” AND “interoception”  “eating disorders” AND “interoceptive”  “eating disorders” AND “body schema”  “eating disorders” AND “body perception” | 26  50  4  7  10  213  16  67 |

| Database | No of records | Duplicates per database | Total items | Non Peer reviewed | Final items |
| --- | --- | --- | --- | --- | --- |
| PubMed | 270 | 71 | 199 | 0 | 199 |
| PsycINFO | 578 | 177 | 401 | 97 | 304 |
| Total | 848 | 248 | 600 | 97 | 503 |
